# Influence of oxytocin administration on somatosensory evoked magnetic fields induced by median nerve stimulation during hand action observation in healthy male volunteers

**DOI:** 10.1371/journal.pone.0249167

**Published:** 2021-03-31

**Authors:** Yasuki Ono, Tetsu Hirosawa, Chiaki Hasegawa, Takashi Ikeda, Kiwamu Kudo, Nobushige Naito, Yuko Yoshimura, Mitsuru Kikuchi

**Affiliations:** 1 Department of Neuropsychiatry, Graduate School of Medicine, Hirosaki University, Hirosaki, Japan; 2 Department of Psychiatry and Neurobiology, Graduate School of Medical Science, Kanazawa University, Kanazawa, Japan; 3 Research Center for Child Mental Development, Kanazawa University, Kanazawa, Japan; 4 Ricoh Company, Ltd, Kanazawa, Japan; Chiba Daigaku, JAPAN

## Abstract

Watching another person’s hand movement modulates somatosensory evoked magnetic fields (SEFs). Assuming that the mirror neuron system may have a role in this phenomenon, oxytocin should enhance these effects. This single-blinded, placebo-controlled, crossover study therefore used magnetoencephalography (MEG) to investigate SEFs following electrical stimulation of the right median nerve in 20 healthy male participants during hand movement observation, which were initially presented as static images followed by moving images. The participants were randomly assigned to receive either oxytocin or saline during the first trial, with the treatment being reversed during a second trial. Log-transformed ratios of the N20 and N30 amplitudes were calculated and compared between moving and static images observations. Phase locking (calculated using intertrial phase coherence) of brain oscillations was also analyzed to evaluate alpha, beta and gamma rhythm changes after oxytocin administration. Log N30 ratios showed no significant changes after placebo administration but showed a decreasing tendency (albeit not significant) after placebo administration, which may suggest mirror neuron system involvement. In contrast, log N20 ratios were increased after placebo administration, but showed no significant change after oxytocin administration. Interestingly, the gamma band activity around N20 increased after placebo administration, suggesting that oxytocin exerted an analgesic effect on median nerve stimulation, and inhibited the gamma band increase. Oxytocin might therefore modulate not only the mirror neuron system, but also the sensory processing associated with median nerve stimulation.

## Introduction

Somatosensory inputs from the periphery reach the contralateral primary somatosensory cortex (SI), which subsequently activates the ipsilateral SI and bilateral secondary somatosensory cortex (SII) [1,2]. Components of the SEF are modulated during the preparatory period for voluntary movement, with some reports showing a crossmodal interaction between somatosensory and visual information such as M35 (which may correspond to N30) amplitude attenuation just before movement [3]. In particular, studies have shown that viewing another person’s gestures modulates excitability in somatosensory areas [4,5]. Rossi et al. [5] demonstrated an increase in the amplitude of the N30 component when a subject watched someone else performing, but showed no change in the amplitude of N20 generated in the posterior wall of the central sulcus (area 3b). The increase in the amplitude of N30, which was localized to the anterior wall of the central sulcus [6], was thought to have been associated with mirror neurons [5,7]. Studies have suggested that the mirror neuron system plays an important role in recognizing actions and understanding their meaning [8]. This system, which is active when either observing or executing the same action is thought to be closely connected to the electrophysiological mu suppression signal [9]. The mu frequency band (8–13 Hz) is typically suppressed during action observation or execution conditions. A recent study with a sequential EEG-functional magnetic resonance imaging (fMRI) design showed similar patterns of mirror neuron activity and mu suppression; however, mu suppression was not confined to the mirror neuron areas, but involved a range of subcortical areas related to motor preparation and visual sensitivity [10].

Increases in the N30 amplitude have been shown to be correlated with decreases in the 20- Hz poststimulus rebound in the anterior wall of the central sulcus, suggesting a similar precentral origin. Using a similar task, Muthukumaraswamy showed decreased mu and beta band power following median nerve stimulation during electroencephalogram wavelet analysis, and suggested a functional role of the mirror neuron system [11]. Cebolla and colleagues [7] applied standardized weighted low-resolution electromagnetic tomography (i.e., images of standardized current density with zero localization error) [7] to localize N30 sources implicated in the time-frequency domain at rest and during observation of another person’s hand movement. Accordingly, they also found that the increase in N30 amplitude correlated with central and precentral alpha and parietal beta phase locking of ongoing electroencephalography (EEG) signals.

Given the lack of studies using somatosensory evoked fields (SEFs) to evaluate the effects of oxytocin on alpha, beta and gamma waves, as well as on the amplitude of N30 during movement observation, the current study sought to evaluate the influence of oxytocin administration on SEF during hand movement observation.

Oxytocin, a neuropeptide hormone that plays an important role in parturition and lactation [12], has recently been the focus of much attention for its prosocial effects and use in the treatment of many mental disorders, including autism [13], schizophrenia [14], and posttraumatic stress disorder [15]. Although the mechanisms through which oxytocin exerts its effects have yet to be fully understood, studies have shown that it could be related to anxiety reduction, affiliative motivation, and perceptual selectivity [16]. However, only a few studies in normal volunteers [17,18], combat veterans under threat [19], and schizophrenia [20] have used mu suppression to evaluate the effects of oxytocin on the mirror neuron system.

In one of these studies, Perry and colleagues [9] demonstrated that oxytocin differentially modulated EEG rhythm in the alpha/mu and beta ranges during tasks involving biological and nonbiological motion in normal subjects. In men with schizophrenia, oxytocin was shown to modulate mu suppression while they were viewing biological motion [14]. Perry et al. [9] suggested that mu and beta suppression over the sensory-motor regions reflected a resonance system in the human brain analogous to mirror neuron activity.

Therefore, we hypothesized that oxytocin would increase the N30 amplitude during hand movement observation, and enhance alpha and beta suppression during hand movement.

## Material and methods

### Participants

A total of 20 right-handed adult men (mean age, 31.3 years; range, 19–46 years) were enrolled, and were the same individuals who participated in our previous study [21] The reason for examining only one sex was to avoid potential interaction of oxytocin with the female hormonal cycle and sex- and age-related differences in response to oxytocin. All participants were native Japanese with no previous or existing psychiatric, neurological, or medical disorders. To ensure that no history of psychiatric illness existed, the participants were screened for DSM-IV-TR Axis I Disorders using structured clinical interviews. All participants provided written informed consent prior to enrollment. The current study was approved by the ethics committee of Kanazawa University Hospital (No 759) and was conducted in accordance with the principles of the Declaration of Helsinki. Autistic tendencies in the participants were evaluated using the Autism Spectrum Quotient, Empathy Quotient, and Systemizing Quotient instruments. The Autism Spectrum Quotient, a validated measure of autism spectrum characteristics found in the general population and in individuals diagnosed with autism, consists of five subscales: social skill, communication, imagination, attention to detail and attention switching [22]. The Empathy Quotient quantifies individual differences in empathizing [23], and the Systemizing Quotient measures individual differences in systemizing [24], which is the cognitive style complementary to empathizing.

### Experimental design and stimuli

Experimental sessions were conducted using a single-blinded, placebo-controlled, within-subject, crossover design, with an interval of at least 2 weeks between the two sessions. For the initial session, participants were randomly assigned to either the experimental condition, or the placebo condition using random sampling numbers. They first completed a task that included median nerve stimulation and SEF measurement while watching video clips of either a simple hand movement or no movement. The participants in the experimental group then received a single intranasal dose (24 IU) of oxytocin (Syntocinon; Novartis, Basel, Switzerland), administered according to gold standard methods in human oxytocin research [25], whereas those in the placebo group received intranasal saline. Both oxytocin and placebo were colorless and transparent. The task was performed again 50 min after oxytocin or placebo administration with the time interval based on published pharmacokinetics data for oxytocin [26]. The participants then returned for a second session, during which the tasks from the first session were repeated but in the other condition (placebo or oxytocin). The mean interval between the two sessions was 73.8 (SD, 56.8) days.

**Fig 1:**
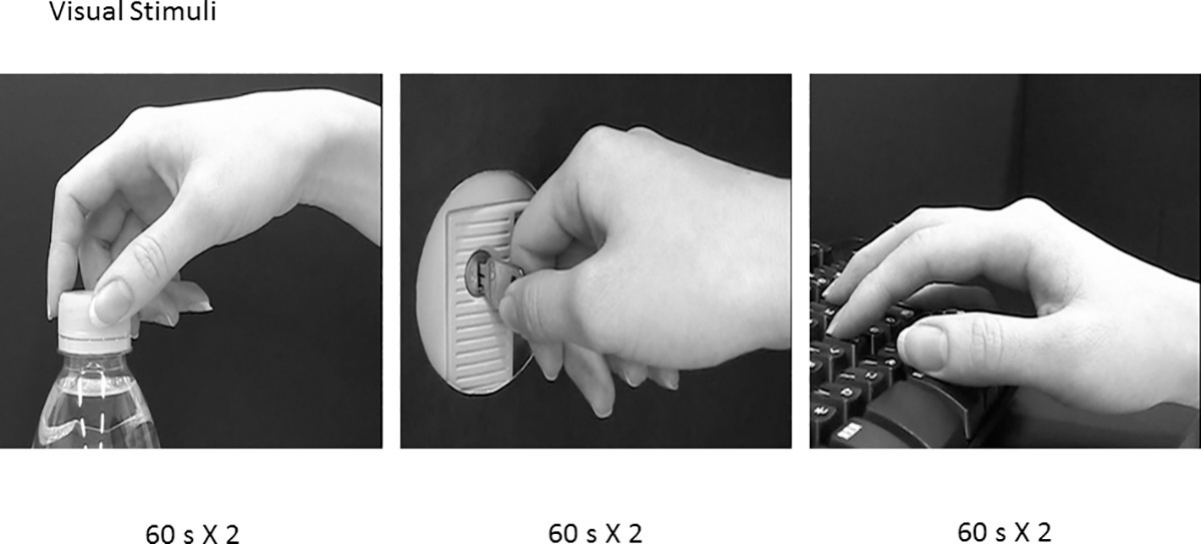
Sample of the images shown in the experiment. From left to right: Opening a bottle, unlocking a lock with a key, and typing on a keyboard.

The experiment was conducted in a magnetically shielded room (Daido Steel, Nagoya, Japan). During the session, the participant lay supine on a bed facing a tilted white screen (24 x 14 cm) approximately 30 cm above their head. Using a video projector (PG-B10S, Sharp, Osaka, Japan), computer images were projected onto the screen at a refresh rate of 60 Hz with the visual angle of the image on the screen being approximately about 42 x 34^o^. Given that each trial lasted 60 s and was repeated twice in the same order, the entire procedure lasted 360 s. For the first 30 s, the participant was shown static images of a model’s right hand opening a bottle, unlocking a lock with a key, and typing on a keyboard (static state; Fig 1). For the next 30 s, the same actions were shown as moving images (motion state). The visual tasks were created using SuperLab 4.0 (Cedrus, San Pedro, CA, USA). While participants watched the static and moving images, median nerve stimulation was performed by electrically stimulating (2 Hz) the right median nerve at the wrist, with an average time randomization of 50%. The SEF was then measured while the participants watched the hand movements.

### Magnetoencephalography recordings and evoked potential

Magnetic fields were measured using a whole-head system for adults at the Laboratory of Yokogawa Electric Corporation in Japan (MEG Vision PQA160C; Yokogawa Electric Corporation, Yokogawa, Japan), which comprises 160 sensors. Magnetic fields were sampled at 1000 Hz per channel (with a bandpass of 0.16–200 Hz). The single equivalent current dipole (ECD) model was used to estimate the ‘‘center of gravity” of the current sources in the activated cerebral cortex using more than 30 sensors [27]. The three-dimensional location, orientation, and strength of the ECD were obtained from a spherical head model based on MR images of the participants. The first two peaks in the 15 to 50 ms poststimulus epochs in the sensorimotor hand region were analyzed. MegLaboratory 160 software (Yokogawa/KIT, Kanazawa, Japan) was used to estimate the localization of the current sources. Only ECDs with a goodness- of fit >85% of the field variance were accepted for further analysis [28]. Using a Signa Excite HD 1.5-T system (GE Yokogawa), all subjects underwent T1-weighted magnetic resonance imaging (MRI) with spherical lipid markers placed at the 5 magnetoencephalography (MEG) fiduciary points to enable superimposition of the MEG coordinate system on the MRI data. MRI consisted of 166 1.2 mm sequential slices, with a resolution of 512 x 512 points in a field of view of 261 x 261 mm. After reconstructing the three-dimensional MR images, the best-fit sphere for each participant’s head was determined.

### MEG preprocessing

Brainstorm [29] and MATLAB (The Math Works, Inc., USA) were used to analyze the data. MEG data were sampled at 2000 Hz using a 200- Hz low-pass filter. Ocular and electrocardiogram artifacts were removed through visual inspection and the signal-space projection method. Epochs contaminated by muscle, heartbeat or eyeblink artifacts that contained field amplitudes greater than + 4 pT were excluded from the analysis.

The pattern search function in Brainstorm was used to scan raw data to identify other blinks and compute the average eye-blink topography across MEG sensors. An eye blink was modeled according to the topography of its first component using principal component analysis. In addition to heartbeat activity, the average heartbeat topography was also computed and modeled using the first component of principal component analysis.

The data were segmented from—100 to 300 ms relative to the median nerve stimulus onset and adjusted to a—100 to—0.5 ms prestimulus baseline. Thereafter, muscle artifacts were excluded through visual inspection or the automatic processing procedure in Brainstorm. Moreover, 40 to 240- Hz artifacts related to muscle noise as well as some sensor artifacts were automatically detected and removed. In detail, Brainstorm allowed for five levels of sensitivity (very sensitive to very conservative), with the very conservative sensitivity level being used herein.

### N20 and N30 evoked potentials and statistical analysis

Log- transformed amplitudes of N20 and N30 (logN20 and logN30, respectively) were analyzed during the tasks before and 50 min after placebo or oxytocin administration to reduce individual variations. Ratios were calculated by dividing logN20 (and logN30) during the motion state by logN20 (and logN30) during the static state after which the results were compared between the placebo and oxytocin conditions using two-way repeated-measures analysis of variance (ANOVA). The two within-subject variables were time course (pre-administration vs. post-administration) and treatment (oxytocin vs. placebo). For multiple comparisons in ANOVA, statistical significance was defined as P < 0.025. For post hoc analyses, paired t- tests were used to evaluate the effects of saline and oxytocin before and after administration.

### Spectral analyses and intertrial phase coherence statistics

The signal source was estimated using the weighted minimum norm estimates and computed with the overlapping spheres algorithm based on individual MR images.

The noise covariance matrix for each subject was calculated using the prestimulus baseline period (-100 to—0.5 ms). Regions of interest (ROIs) were determined based on the Desikan-Killiany gyrus atlas segmented by Freesurfer. A time-frequency analysis was performed using Morlet wavelets with a central frequency of 2 Hz, and a time resolution of 3 s for the mother wavelet. Thereafter intertrial coherence (ITC) was computed, by which phase-locking was measured. ITC is a normalized measure assessing trial-to-trial similarity in oscillatory activity, with ITC = 1 reflecting no phase variability and ITC = 0 reflecting maximal phase variability across trials.

To compare neural activation between observation of the static images and moving images, paired t-tests (18 degrees of freedom) were conducted for each ROI within each time frame (from 0 to 300 ms) and for each frequency frame (from 2 to 50 Hz). The threshold for statistical significance was set at p < 0.05, with false discovery rate correction across 68 ROIs and 49 frequency dimensions.

## Results

The participants’ mean Autism Spectrum Quotient, Empathy Quotient, and Systemizing Quotient scores were 14.4 (SD, 4.7), 37.5 (SD, 10.2), and 20 (SD, 11.9), respectively.

**Fig 2:**
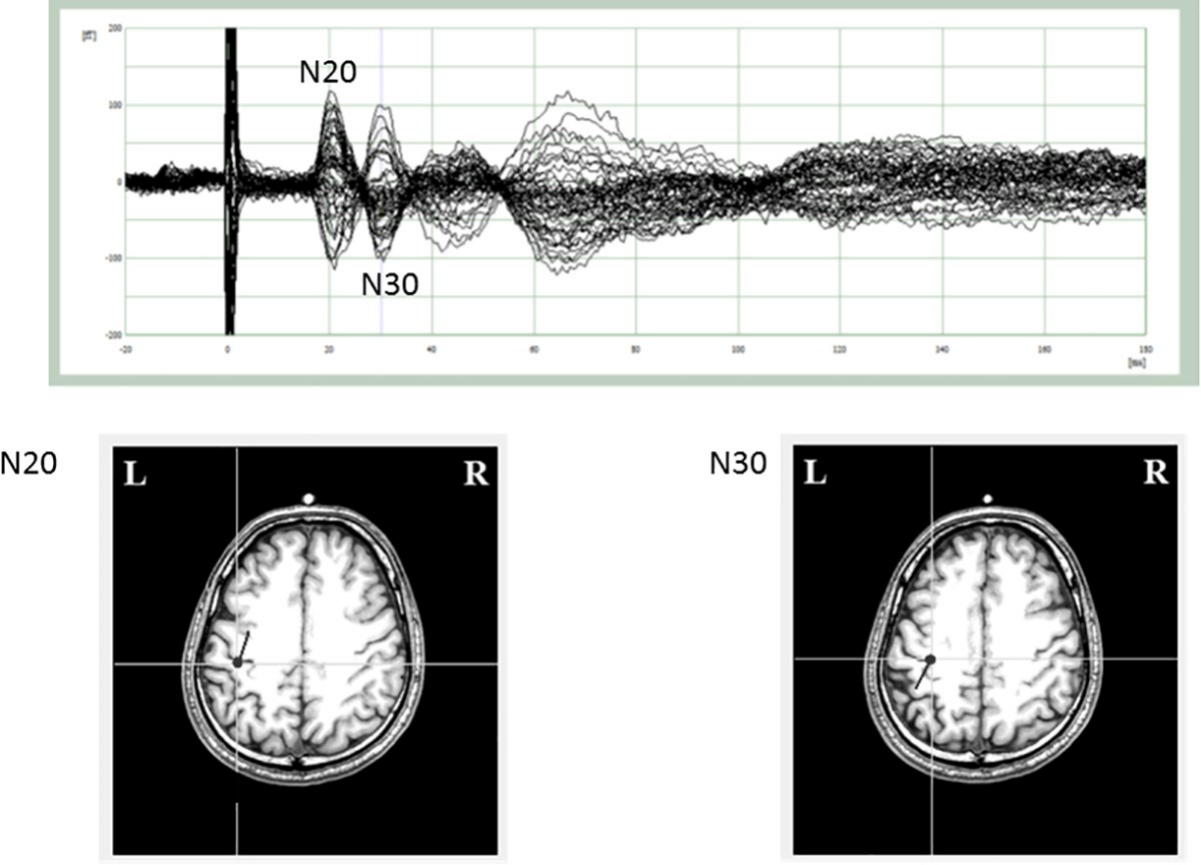
Somatosensory evoked magnetic fields (SEFs) of a representative participant following right median nerve stimulation during hand movement observation. (A) A waveform obtained from several MEG sensors in the primary somatosensory cortex (SI). Two main components, N20 m and N30 m were identified. (B) Localization and direction of the equivalent current dipoles (ECDs) for each SEF component on the magnetic resonance (MR) images. The N20 ECD was located in the posterior bank of the central sulcus in the hemisphere contralateral to the stimulated side, while the N30 ECD was located in the anterior bank of the central sulcus in the hemisphere contralateral to the stimulated side.

Among the 20 participants, who watched the static and moving images, N20 and N30 were detected in 17 and 15, respectively. All detected N20 ECDs were localized in the primary somatosensory cortex (the posterior wall of the central sulcus), while all N30 ECDs were localized in the frontal cortex (the anterior wall of the central sulcus) (Fig 2).

**Fig 3:**
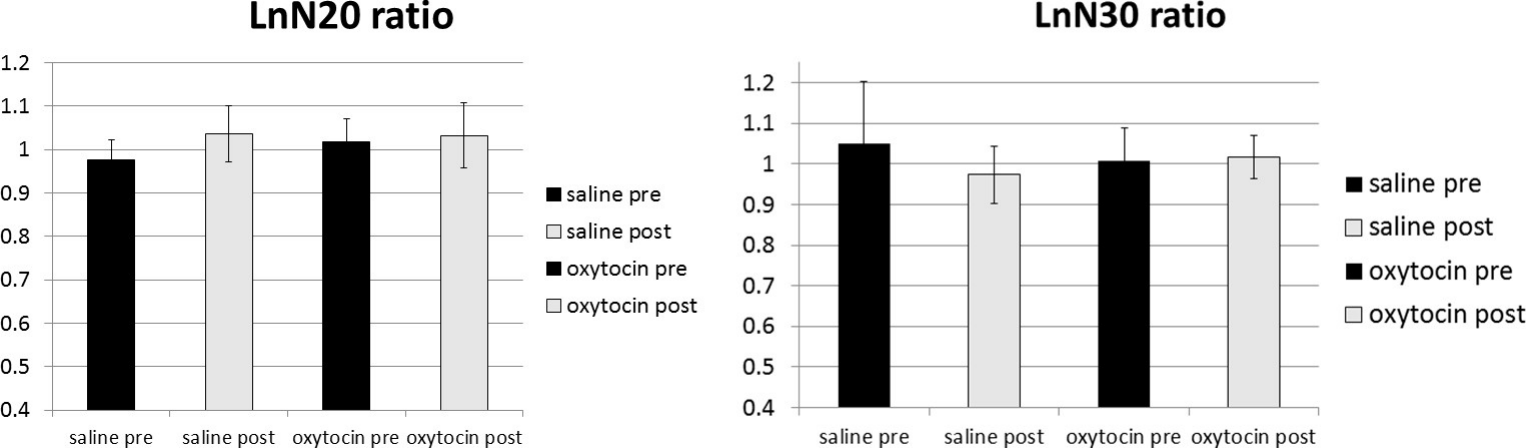
Mean and standard deviation of the equivalent current dipole strength of logN20 m and log N30 m ratios (the ratios of log amplitudes during moving image observation to those during static image observation) before and after placebo or oxytocin administration. The vertical lines indicate the standard deviation (*p < 0.025). (A) ANOVA indicated a significant main effect of time course (preadministration vs. postadministration) for the N20 ratio but no significant main effect of treatment (oxytocin vs. saline) or treatment x time course interaction. (B) There was no significant main effect of treatment or time course, but a significant treatment x time course interaction was observed for the N30 ratio.

Regarding logN20, repeated-measures ANOVA indicated a statistically significant main effect of time course [F (1, 16) = 6.304, p = 0.023], but no significant main effect of treatment [F(1,16) = 1.438, p = 0.248] or interaction of treatment x time course [F(1, 16) = 1.668, p = 0.215] (Fig 3A, Table 1). While a significant increase in logN20 ratios was detected after saline administration (paired t-tests, p = 0.009), no significant change in the logN20 ratio was observed after oxytocin administration (paired t-tests, p = 0.595).

**Table 1:** Sensory evoked fields after saline or oxytocin administration during action observation.

	pre saline	post saline	pre oxytocin	post oxytocin	F1	F2	F1*F2
LnN20 ratio	0.98 (0.05)	1.04 (0.06)	1.02 (0.06)	1.03 (0.08)	0.023*	0.248	0.215
LnN30 ratio	1.05 (0.15)	0.97 (0.07)	1.01(0.08)	1.02 (0.05)	0.287	0.912	0.023*

Two—way repeated—measures ANOVA: F1 = time course; F2 = treatment effect.

F1 * F2 = time course x treatment interaction effect.

Significance set at *p < 0.025.

For log N30 no significant main effect of treatment [F (1, 14) = 0.013, p = 0.912], or time course [F(1, 13) = 1.233, p = 0.287] was observed, but a significant treatment x time course interaction effect [F(1, 13) = 6.668, p = 0.023) was observed (Fig 3B, Table 1). No significant change in log N30 ratios was noted after saline or oxytocin administration (paired t-tests, p = 0.076 and p = 0.691, respectively).

**Fig 4:**
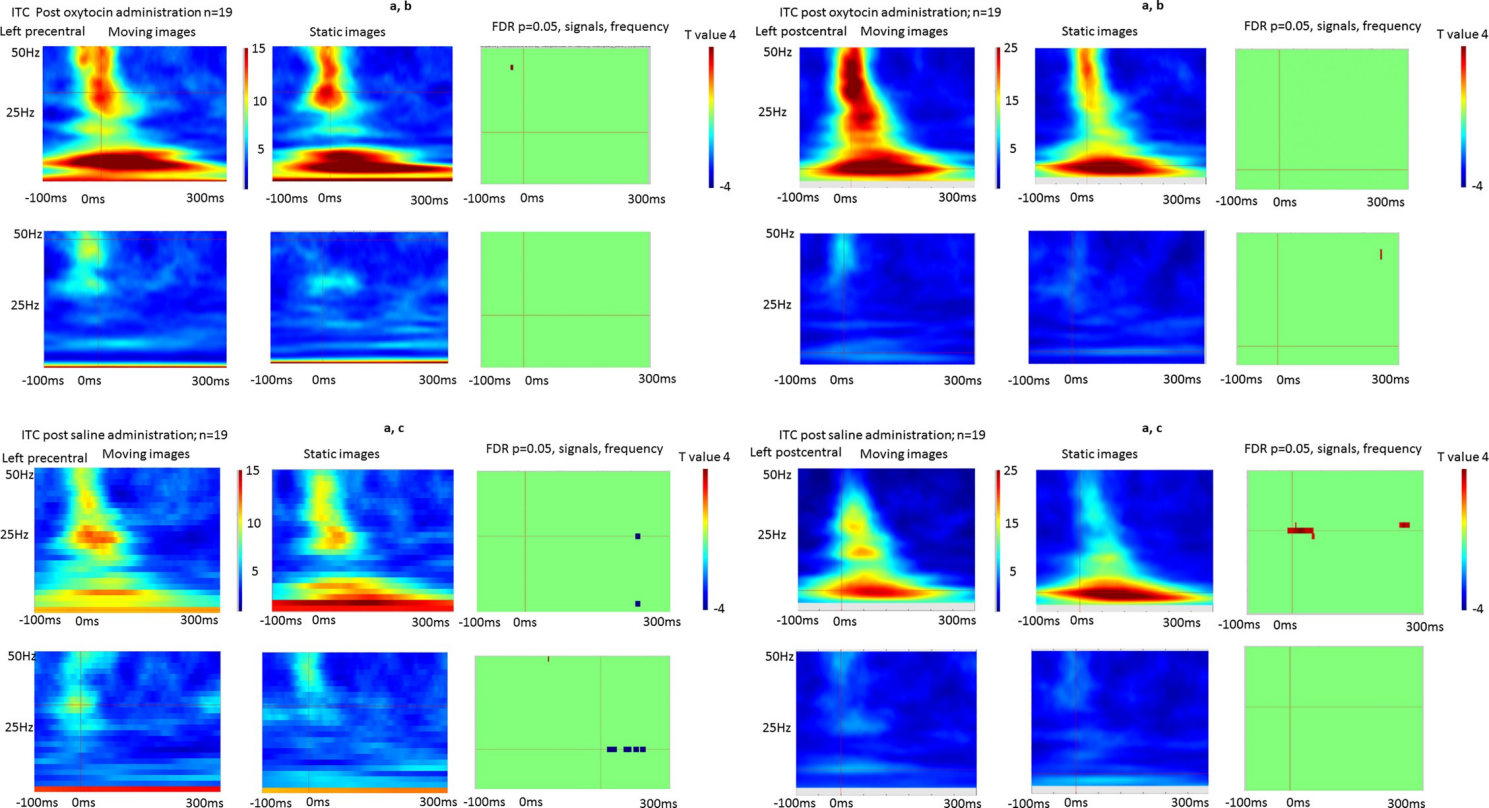
(A) The grand average of the time-frequency maps of Intertrial phase coherence (ITC) during moving and static image observations in the precentral and postcentral gyri after saline or oxytocin administration. (B) T maps of the difference in ITCs between moving and static image observations in the pre and postcentral gyri after oxytocin administration. No significant differences were found in either the precentral or postcentral gyri, when the statistical threshold was set at p < 0.05 with a false discovery rate correction. (C) T maps of the difference in ITCs between moving and static image observations in the precentral and postcentral gyri after saline administration. Significantly lower ITCs were observed in the right precentral gyrus, whereas significantly higher ITCs were observed in the left postcentral gyrus, when the threshold was set at p < 0.05 with a false discovery rate correction.

Fig 4A illustrates the grand average of the ITC for all subjects in the bilateral precentral and postcentral gyri during moving and static images observations after saline and oxytocin administration. The N30 component was accompanied by an increase in gamma phase locking in both the precentral and postcentral gyri on the left hemisphere.

Paired-sample t-tests failed to identify significant ITC differences in the bilateral precentral and postcentral gyri between moving static image observations after oxytocin administration, when the statistical threshold was set at p < 0.05 with false discovery rate corrections across ROIs, signals, and frequency dimensions (Fig 4B). After saline administration, however beta suppression was observed in the right precentral gyrus, while an enhanced gamma band was observed in the left postcentral gyrus, when the statistical threshold was set at p < 0.05 with false discovery rate corrections.

## Discussion

The current study demonstrated that both the logN30 and the logN20 amplitude ratios were influenced by oxytocin administration. Although no statistically significant increase in the logN30 amplitude ratio (the ratio between moving and static images observations) was observed after oxytocin administration, two-way repeated-measures ANOVA showed a significant interaction between time course and treatment, suggesting that oxytocin might act on mirror neurons to induce changes in the logN30 ratio.

Hobson had previously identified the careful consideration of the baseline used for calculation and appropriate sample size as key points for future experiments examining mu suppression [30]. Rossi et al. [5] showed that observing hand movements promoted a larger N30 amplitude than during the rest condition in healthy subjects. In the aforementioned study, the baseline state involved fixating on a stable point on a wall in front of the subject, whereas the observation condition involved watching a repetitive grasping movement and a sequence of finger movements performed with the right hand by one of the examiners. Muthukumaraswamy also used fixation cross observation as the baseline condition [11], whereas Cebolla used an eye- closed condition for the baseline [7].

The current study, which compared stationary and moving images of the same movement, found no difference in the N30 amplitude without oxytocin, which might activate the mirror neuron system. Several studies have previously reported an increase in N30 during hand movement observation, which may be related to the mechanism of storage and encoding somatosensory information connected exclusively with the observed hand movement [7]. In particular, increase in alpha can be explained by top-down inhibitory control processes that improve behavioral performance [31].

Contrary to our secondary hypothesis, the observation of moving images did not produce greater alpha and beta suppression than the observation of static images after oxytocin administration, with the response being similar to that observed before oxytocin and saline administration. However, after saline administration, the observation of moving images induced greater beta suppression in the precentral gyrus of the ipsilateral cortex, and a higher gamma wave increase in the postcentral gyrus of the contralateral cortex than observation of the static images.

Although Perry’s study showed that observation of biological motion after oxytocin administration clearly induced alpha and beta suppression [9], our experiment was different in that the median nerve was simultaneously stimulated.

Regardless of the exact mechanism, there is moderate evidence, especially from the animal literature, to suggest that oxytocin exerts analgesic effects [32]. One mechanism is a direct hypothalamo-spinal projection from the paraventricular nucleus of the hypothalamus to the dorsal horn of the spinal cord [33]. Another mechanism involves the relationship between oxytocin and the endogenous opioid system [34].

Thus, the influence of oxytocin on sensory pain warrants consideration. N20 is the earliest SEF deflection from the primary sensory cortex and is involved in discriminative aspects of sensory information such as intensity [35]. The present study found an increase in the log N20 amplitude ratio after saline administration, but no significant increase after oxytocin administration. Oxytocin has been found to have analgesic effects and reduce the local peak amplitude using laser-evoked potentials [36]. As such, the attenuation of median nerve stimulation and lack of an increase in the log N20 ratio can be attributed to oxytocin’s analgesic effects. To support this notion, our study showed that gamma band activity was increased after saline administration but not after oxytocin administration. Other studies have found that somatosensory induced gamma oscillations emerged in the postcentral gyrus after median nerve stimulation [37] and that the strength of the gamma power increased with increasing stimulus intensity [38].

Weak beta suppression was observed in the ipsilateral precentral cortex. Although alpha power has been predominantly linked to the somatosensory cortex, beta suppression has been more related to motor processing and the primary cortex [39]. This may explain why only beta suppression was detected in the precentral cortex in this study.

In previous studies, the stimulation intervals were different from those in our study; alpha suppression has been shown to begin around at approximately 300–700 ms [5,11,40]. In our study, stimulation was 2 Hz and we analyzed the data from -100 to 300 ms. Therefore, this may have to be too short of a duration to detect alpha rebound.

Mu rhythm suppression has been used to measure mirror neuron activity, but there is not enough evidence to demonstrate the specificity of mu rhythm.

Coll et al. [41] evaluated mirror neuron activity using cutaneous tactile stimulation during action observation.

They used a multivariate analytical approach to show crossmodal EEG mu rhythms with traditional univariate analysis (paired t-test and ANOVA). They showed that traditional univariate analysis was insensitive for monitoring mu rhythm activity. Therefore, we consider this a limitation of our analytical method.

Feedforward connectivity is defined as connections that arrive in layer 4, the granular layer in the cortex, and progress upward along the cortical hierarchy [42]. In the present study, feedforward activity would correspond to direct interference between the visual input related to the movement and an afferent somatosensory volley triggered by that movement. Feedback connectivity refers to inputs that arrive in the supragranular layers of the cortex and usually progress downward or in parallel across the cortical hierarchy [42]. A feedback mechanism would correspond to a change in the functional state of the network, induced by the context of performing an observation [43]. In autism spectrum disorder, the long-range feedforward (bottom-up) connectivity is abnormally increased, whereas feedback (top- down) long range connectivity is abnormally reduced [43]. Given that N30 has a considerably short latency, direct interference between the visual input from observing hand movement and the somatosensory input can be neglected [7]. N30 modulation occurs before the arrival of the visual afferent volley related to somatosensory stimulation [7]. The top- down influence of the mirror neuron system can be modulated by oxytocin.

Some limitations associated with the present study warrant consideration. First, this study had no control condition against which the observation task could be compared. Thus, only differences between static and moving images could be evaluated. Second, given that the stimulator produced some stimulus artifacts, it was sometimes difficult to detect the trigger for median nerve stimulation, which resulted in some unsteadiness in the SEF. Third, this study used saline as a placebo. Considering that oxytocin might occasionally induce a sour taste on the tongue, some differences between placebo and oxytocin might have been present.

## Conclusions

The current study demonstrated that the ratio of the log-transformed N20 amplitudes between observing moving and static images of hand movements increased after saline administration accompanied by a gamma band increase in the post central region. It appeared that oxytocin decreased the gamma band activity and counteracted the increase in the logN20 ratio. The equivalent ratio with N30 amplitude differed between oxytocin and saline administration, suggesting mirror neuron system involvement. Contrary to our hypothesis, oxytocin did not induce alpha and beta suppression during hand movement observation but might have analgesic effects.

## Supporting information

S1 Fig Interval between the first- and second- day. No significant correlations were observed between the first and second day intervals and the logN20 and N30 ratios.
(TIF)

S2 Fig Region of interest for the single equivalent current dipole. (TIF)

S3 Fig Grand average of the event- related spectral perturbation (ERSP) for all subjects in the bilateral precentral and postcentral gyri during moving and static image observation after saline and oxytocin administration. The N30 component was accompanied by an increase in gamma phase locking in both the precentral and postcentral gyri in the left hemisphere. Paired-sample t-tests failed to identify significant differences in the ERSP in the bilateral precentral and postcentral gyri between moving and static image observations after oxytocin administration when the statistical threshold was set at p < 0.05 with false discovery rate corrections across ROIs, signals, and frequency dimensions. No significant differences were found in either the precentral or postcentral gyri when the statistical threshold was set at p < 0.05 with a false discovery rate correction.
(TIF)
