# Supplementary figures and images for: Influence of oxytocin administration on somatosensory evoked magnetic fields induced by median nerve stimulation during hand action observation in healthy male volunteers
Source: PLoS One. 2021 Mar 31;16(3):e0249167. doi: 10.1371/journal.pone.0249167 (PMC8011787; doi:10.1371/journal.pone.0249167)

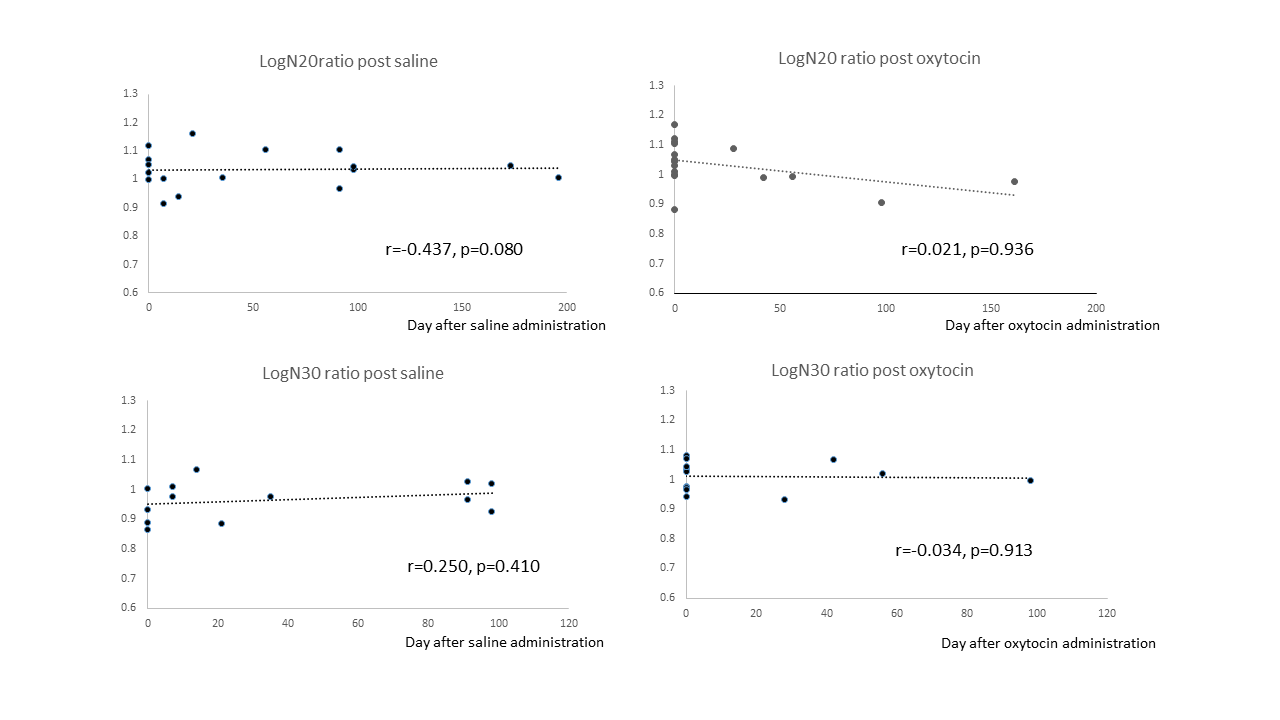

Supplement: S1 Fig — No significant correlations were observed between the first and second day intervals and the logN20 and N30 ratios. (TIF) [file pone.0249167.s001.tif]

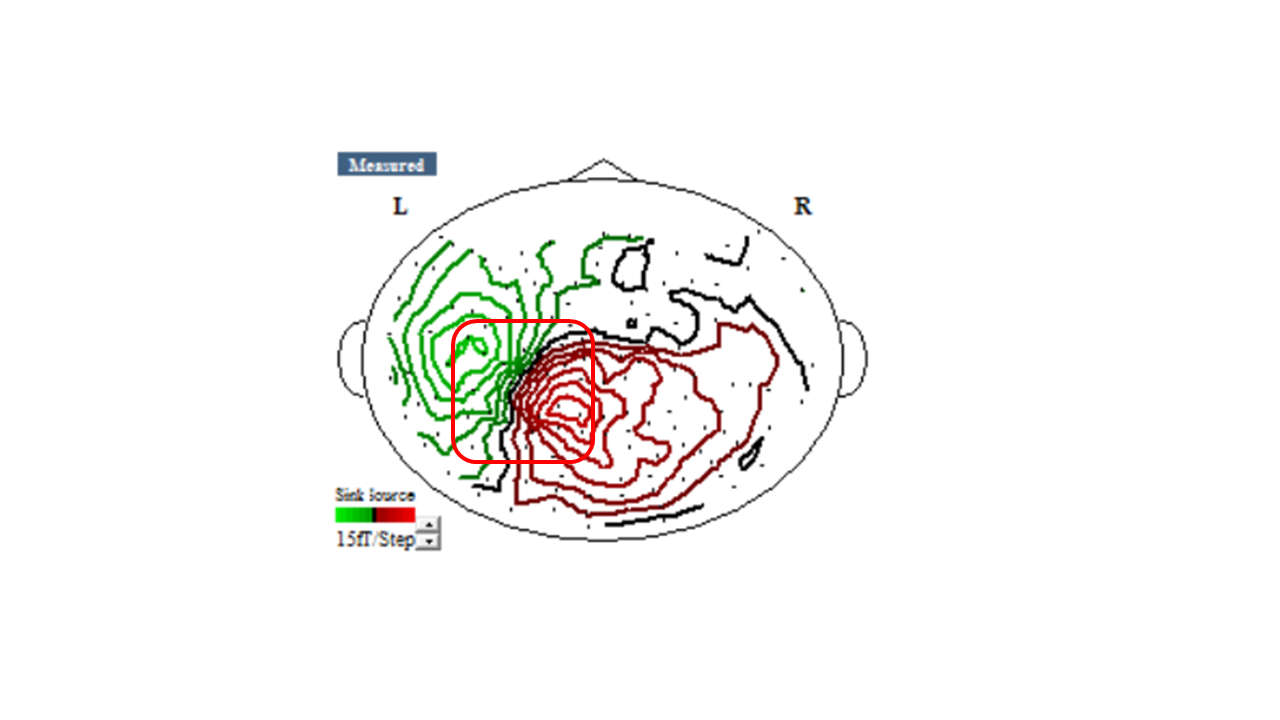

Supplement: S2 Fig — (TIF) [file pone.0249167.s002.tif]

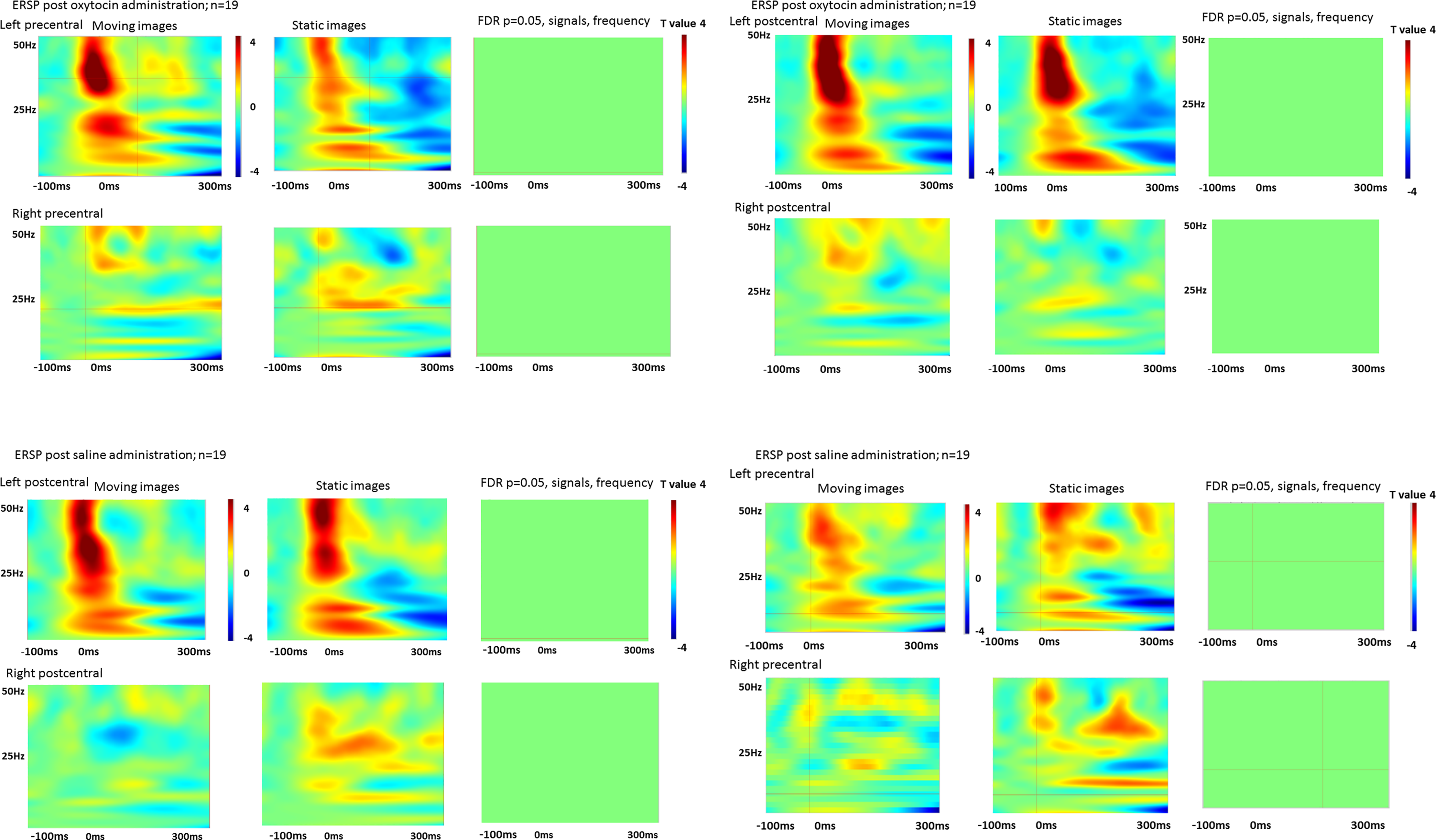

Supplement: S3 Fig — The N30 component was accompanied by an increase in gamma phase locking in both the precentral and postcentral gyri in the left hemisphere. Paired-sample t-tests failed to identify significant differences in the ERSP in the bilateral precentral and postcentral gyri between moving and static image observations after oxytocin administration when the statistical threshold was set at p < 0.05 with false discovery rate corrections across ROIs, signals, and frequency dimensions. No significant differences were found in either the precentral or postcentral gyri when the statistical threshold was set at p < 0.05 with a false discovery rate correction. (TIF) [file pone.0249167.s003.tif]
